# Brain and brain-heart Granger causality during wakefulness and sleep

**DOI:** 10.3389/fnins.2022.927111

**Published:** 2022-09-15

**Authors:** Helmi Abdalbari, Mohammad Durrani, Shivam Pancholi, Nikhil Patel, Slawomir J. Nasuto, Nicoletta Nicolaou

**Affiliations:** ^1^Department of Basic and Clinical Sciences, University of Nicosia Medical School, Nicosia, Cyprus; ^2^Department of Biomedical Engineering, School of Biological Sciences, University of Reading, Reading, United Kingdom; ^3^Center for Neuroscience and Integrative Brain Research (CENIBRE), University of Nicosia Medical School, Nicosia, Cyprus

**Keywords:** sleep, electroencephalogram (EEG), electrocardiogram (ECG), Granger causality, connectivity

## Abstract

In this exploratory study we apply Granger Causality (GC) to investigate the brain-brain and brain-heart interactions during wakefulness and sleep. Our analysis includes electroencephalogram (EEG) and electrocardiogram (ECG) data during all-night polysomnographic recordings from volunteers with apnea, available from the Massachusetts General Hospital’s Computational Clinical Neurophysiology Laboratory and the Clinical Data Animation Laboratory. The data is manually annotated by clinical staff at the MGH in 30 second contiguous intervals (wakefulness and sleep stages 1, 2, 3, and rapid eye movement (REM). We applied GC to 4-s non-overlapping segments of available EEG and ECG across all-night recordings of 50 randomly chosen patients. To identify differences in GC between the different sleep stages, the GC for each sleep stage was subtracted from the GC during wakefulness. Positive (negative) differences indicated that GC was greater (lower) during wakefulness compared to the specific sleep stage. The application of GC to study brain-brain and brain-heart bidirectional connections during wakefulness and sleep confirmed the importance of fronto-posterior connectivity during these two states, but has also revealed differences in ipsilateral and contralateral mechanisms of these connections. It has also confirmed the existence of bidirectional brain-heart connections that are more prominent in the direction from brain to heart. Our exploratory study has shown that GC can be successfully applied to sleep data analysis and captures the varying physiological mechanisms that are related to wakefulness and different sleep stages.

## Introduction

Sleep is a complex physiological process, with an essential role in maintaining normal cognitive functions and facilitating memory processes. The process of sleep affects the entire body, which is an integrated network of sub-systems, each with their own internal regulatory mechanisms, interacting with each other to maintain normal physiological functions. Sleep is sub-classified into two main types: non-rapid eye movement (NREM) and rapid eye movement (REM) sleep. The former is subdivided further into three stages: relaxed wakefulness (S1), light-sleep (S2) and slow-wave deep sleep (S3) (Nayak and Anilkumar, 2022). Each stage is characterized by different physiological activity in the central and autonomic nervous systems, but a common way of identifying and classifying sleep into different stages utilizes the distinct EEG patterns observed in each one. In the awake stage, EEG is characterized by fast low-voltage frequencies, also known as beta rhythm (13–24 Hz). As we transition to falling asleep and NREM sleep dominates, beta waves are progressively substituted by slower frequency waves. Before the commencement of S1 stage, alpha waves (8–12 Hz) with some beta waves appear. S1 shows the disappearance of the alpha waves with medium frequency amplitude waves taking over. During this stage, alerting the person can lead to alpha wave recurrence. S2 shows appearance of K-complexes, which have a V-shaped pattern, alongside theta waves (4–7 Hz). S3 is characterized by changes from theta waves to the slower and larger delta waves (1–4 Hz). Following the NREM stage, REM sleep occurs, which has characteristic saw-tooth waves intermittently with low-voltage, random fast waves similar to the beta waves seen when awake (Nayak and Anilkumar, 2022).

Even though sleep affects the entire brain, it does not necessarily begin simultaneously in all cortical areas. It has been shown that ‘*sleep is not only a global phenomenon but also a local brain process with a different regional involvement of neuronal populations’* (Werth et al., 1997). This is also evident in changes in brain connectivity that have been found to characterize different sleep stages. During light sleep (N2), the increase in interdependent EEG signals is related to increased temporal synchronizations. As an example, as we progress into deeper stages of sleep, Synchronization Likelihood (SL) is reduced, indicating reduction in recurrence of temporal signal patterns. By the time the stage of sleep gets into REM, the brain presents with more interdependent signals that decrease temporal synchronicity at this stage, the brain has more complex interactivity (Migliorelli et al., 2009). There is also a functional dissimilarity between the REM and NREM sleep stages (Dimitriadis et al., 2008) as slow wave activity occurs predominantly in NREM, which is caused by quick alterations in hyperpolarized and polarized states of neurons (Valderrama et al., 2012). As a result, the disintegration of brain complexity occurs due to the inability of the thalamocortical system to engage in complex patterns (Sarasso et al., 2014). Using EEG with concurrent transcranial magnetic stimulation (TMS), it has been found that effective connectivity breaks down during NREM sleep compared to quiet wakefulness (Massimini, 2005). Regarding the state of dreaming or REM sleep stage, the recovery of conscious experience with cortical pattern activation is similar to wakefulness (Sarasso et al., 2014).

Growing evidence support that sleep EEG is characterized by wide frequency-specific and state-specific differences across the fronto-posterior brain axis (Werth et al., 1997; Ferrara et al., 2002). In the study of Lee et al., higher Phase-Locking Value (PLV) was observed between electrodes in the alpha and beta bands in conscious experience compared to no conscious experience with topographical analysis revealing a higher clustering coefficient in parietal-occipital regions in delta band in non-conscious experience than conscious experience (Lee et al., 2009). Describing the transition from wakefulness to sleep, the propagation of nerve signals is such that during deep sleep, slow oscillations sweep the cortex in an antero-posterior direction (Massimini et al., 2004). Slow rhythms are more prominent in the fronto-parietal lobes compared to the occipital lobes which supports the notion that spread of synchronizing signals from associative pre-frontal zones to posterior zones have an important role in wakefulness-sleep transition (De Gennaro et al., 2004).

Other measurements of brain function that have been used in the study of the awake-sleep cycle include blood oxygen level dependency detected from functional magnetic resonance imaging (fMRI), to look at connections within the brain during wakefulness and sleep. In a study by Lv et al., graph theory was used to analyze such data during wakefulness and sleep, and found that there was decreased connectivity between the paralimbic-limbic cortex and the neocortical system and the centrencephalic structure during sleep (Lv et al., 2015). This finding shows that there is a suppression in the interference of the external environment within the brain during sleep as compared to wakefulness, supporting the existence of a ‘defense mechanism’ in place to prevent external interference during sleep. Another study looked into using single pulse electrical stimulation to monitor differences in brain connectivity during sleep as compared to wakefulness (Usami et al., 2019). An increased propagation to the parietal lobe was found during slow wave sleep when the frontal lobe was stimulated as compared to wakefulness. During REM sleep it was found that there was an increase in parietal lobe propagation and a decrease in frontal lobe propagation when stimulated, respectively.

In addition to changes in central nervous system activity, the autonomic nervous system (ANS) controls and regulates many biological processes during sleep. As the transition happens between wakefulness and NREM sleep, the parasympathetic drive increases while the sympathetic drive decreases. This trend continues along the transition from NREM to REM sleep with the only exception being in phasic REM sleep (where there is a slight increase in the sympathetic tone) (Lee-Chiong, 2008). During NREM sleep, there is a drop in the heart rate as well as a decline in the mean arterial pressure by 10% compared to that of wakefulness. This is known as the dipping phenomenon and can be attributed to the decrease in sympathetic drive to the heart (Silvani and Dampney, 2013). The nucleus of the solitary tract located in the medulla, is what regulates the sympathetic outflow to the heart (Andresen et al., 2006). Furthermore, circadian rhythm changes are believed to be the reason behind the increase in parasympathetic effects on cardiac activity (Burgess et al., 1997), an example of these changes is night time reduction in catecholamines levels (Lee-Chiong, 2008). Along with cardiovascular changes during sleep, there is a drop in the respiratory rate. Respiration is driven metabolically during sleep and the controlling factor is the Bötzinger complex, a cluster of neurons located in the rostral ventrolateral medulla. The Bötzinger complex receives projections form the nucleus of the solitary tract (Spyer and Gourine, 2009).

Heart rate variability (HRV) is a universally accepted term used to describe both the variations in heart rate and R to R (RR) intervals (Task Force of the European Society of Cardiology and the North American Society of Pacing and Electrophysiology, 1996). HRV changes during different stages of the sleep wake cycle and HRV data correlates strongly with the hypothesis that adverse cardiovascular events occur predominantly early in the morning shortly after awakening. This is because the sleep-to-wake cycle transition in the morning has the highest activation towards the sympathetic nervous system compared to the rest of the day. This is shown by the increased low frequency to high frequency ratio (LF:HF), which is interpreted as an increase in the sympathovagal balance (Boudreau et al., 2013). Changes in autonomic function, which are mirrored in HRV (Kamen et al., 1996), can be evaluated through the use of Poincaré plots (Ardissino et al., 2019). Poincaré plots provide non-linear, geometrical representations of HRV dynamics over a period of time. The width (SD2) of the Poincaré plot reflects parasympathetic activation, while its length (SD1) reflects sympathetic antagonism to vagal tone. Furthermore, the SD1/SD2 ratio is analogous to the spectral measure of LF/HF ratio, indicating sympathovagal balance. Despite the popularity of time-frequency HRV analysis, it has been shown that this is susceptible to high levels of respiratory noise (Penttilä et al., 2001). On the contrary, Poincaré plots are not as susceptible to respiratory noise compared to other methods of HRV analysis, while correlating directly with spectral data (Brennan et al., 2001).

Granger Causality (GC) is another measure that can be used to capture bidirectional connectivity not only in a single-system (EEG), but also across systems (e.g., EEG–ECG). Despite the popularity of GC in neuroscience applications, including in the study of physiological states similar to sleep, such as anesthesia (Nicolaou et al., 2012; Pullon et al., 2020), there are only a handful of studies applying it in sleep. An example is the study by Hartmann et al., where GC was used to study the brain-heart interactions during the cyclic alternating pattern of non-rapid eye movement sleep (Hartmann et al., 2021). According to the authors, their study provided the first evidence *on the causal interplay between cortical and cardiovascular activities during cyclic alternating pattern*. Faes et al., also present brain-heart causality across different frequency ranges during sleep in healthy subjects (Faes et al., 2015, 2014). In this exploratory study we apply GC to investigate the differences in brain (EEG) and brain-heart (EEG-ECG) connectivity during wakefulness and sleep, in patients with sleep apnea. We hypothesize that GC will be able to capture physiological mechanisms of brain-brain and brain-heart changes that characterize wakefulness and sleep, as well as provide complementary information to measures that are commonly applied to sleep analysis (e.g., non-directional correlation, coherence etc.).

## Materials and methods

### Dataset

The data used in this study is available online via PhysioNet (Goldberger et al., 2000), as part of the “You Snooze You Win - The PhysioNet Computing in Cardiology Challenge 2018” [(Ghassemi et al., 2018); see Data availability statement for access details]. The data has been collected during polysomnographic sleep studies to detect sources of arousal (non-apnea) during sleep and was provided by the Massachusetts General Hospital’s (MGH) Sleep Lab, the Computational Clinical Neurophysiology Laboratory and the Clinical Data Animation Centre. The entire dataset comprises 1,985 subjects monitored at the MGH Sleep Lab for the diagnosis of sleep disorders. A variety of physiological signals were recorded as the subjects slept through the night: EEG, ECG, electrooculogram, electromyogram, and oxygen saturation. Signals were sampled at 200 Hz (with the exception of oxygen saturation, which was subsequently resampled to 200 Hz). The EEG was recorded using the sleep EEG montage recommended by the American Academy of Sleep Medicine at 6 standard international 10/20 placement locations with mastoid references (M1, M2): F3-M2, C3-M2, O1-M2, F4-M1, C4-M1, and O2-M1. ECG was recorded below the right clavicle near the sternum and over the left lateral chest wall. Annotations of 30-second segments under one of five sleep stages (wakefulness, stage 1, stage 2, stage 3, and REM) and “undefined” were made by clinical staff at the MGH, according to the American Academy of Sleep Medicine manual for the scoring of sleep. As the dataset was provided as part of a competition challenge, sleep stage annotations are available for only half of the records (994). In this study we analyzed EEG and ECG data from 50 randomly chosen subjects extracted from the records that are annotated.

### Granger causality

Initially a notion introduced by Wiener (1956), it was later formalized by Granger (1969) in what is today known as GC. It is used to infer the directionality and dynamics of influence between two (or more) sources. In its simplest definition, a process, X, is said to Granger-cause another process, Y, if the past of X assists in the prediction of the present of Y beyond the degree by which the present of Y is predicted by the past of Y alone. The traditional formulation relies on the use of autoregressive (AR) models of order *p* for the linear prediction of each variable, **X** =[**x**(**1**),**x**(**2**),…,**x**(**T**)] and **Y** =[**y**(**1**),**y**(**2**),…,**y**(**T**)] (where *T*: number of time-series samples) using information from its own past only (univiate AR – Equation 1) or using information from the past of both variables (bivariate AR – Equation 2). The GC is then estimated by comparing the variance of the residuals from the two models (Equation 3).


(1) $$x\,\left(t\right)=\sum_{i=1}^{p}a_{i}\,x\,\left(t-i\right)+\varepsilon_{x}\,\left(t\right)$$

(2) $$x\,\left(t\right)=\sum_{i=1}^{p}b_{i}\,x\,\left(t-i\right)+\sum_{i=1}^{p}c_{i}\,y\,\left(t-i\right)+\varepsilon_{x\,y}\,\left(t\right)$$

(3) $$GC\left(y\to x\right)=ln\frac{v\,a\,r\,\left(\varepsilon_{x}\right)}{v\,a\,r\,\left(\varepsilon_{x\,y}\right)}$$

In the above equations, *a* is the estimated univariate AR model parameter; *b* and *c* are the estimated bivariate AR model parameters; ε_*x*_ (ε_*xy*_) is residual noise for the univariate (bivariate) AR model; and *var*(.) is the variance. Equation 3 is an estimate of the GC in the direction *y*→*x*.

From the GC definition, it can be seen that GC is always positive and represents the amount by which the past of one variable improves the prediction of another variable. If there is significant GC, then it is said that “Y is causal to X,” and vice versa if in the above equations the roles of X and Y are switched. GC becomes zero if there is no improvement in prediction. In this traditional definition of GC it can be seen that causal effects resulting either from direct or indirect relationships with other processes are not taken into account. Thus, the traditional definition of pairwise GC has been extended to include multivariate AR models that include observations from additional variables, non-parametric approaches, as well as models that capture non-linear relationships (Nicolaou and Constandinou, 2016).

In this work we use the multivariate autoregressive (MVAR) GC as implemented in the Matlab^®^ toolbox eGC (Schiatti et al., 2015). The order of the MVAR model was estimated using both the Akaike and Minimum Description Length criteria.

### Analysis methodology

(1) For each subject, the EEG and ECG records were bandpass filtered from 0.1 to 40 Hz, using an FIR filter (filter order 35, Hamming window). The filtered signals were then split into non-overlapping 4-s segments.
(2) Each segment was further pre-processed by demeaning, first order differencing and testing for stationarity (Kwiatkowski-Phillips-Schmidt-Shin test) (Kwiatkowski et al., 1992). Any segment that was not stationary was excluded from further processing.
(3) GC was estimated for each 4-s segment and only GC values that were statistically significant were kept for subsequent analysis. Prior to GC estimation, the AR order, *p*, was estimated for all segments, for *p* = 1,2,…,30. Based on both the Akaike and MDL criteria, an AR order of 20 was chosen for GC estimation.
(4) The average and grand average significant GC (statistical significance estimated at 95% significance level using Fisher F-test, as provided in the eGC toolbox (Schiatti et al., 2015)) corresponding to each sleep stage was obtained for each subject and over all subjects respectively. Segments that corresponded to arousal due to apnoea were not included in the analysis.
(5) To identify differences in GC between the different sleep stages, the GC for each sleep stage was subtracted from the GC during wakefulness. Positive (negative) differences indicated that GC was greater (lower) during wakefulness compared to the specific sleep stage. This allowed us to easily identify underlying changes in GC that took place during sleep compared to wakefulness. The resulting differences were plotted in topographic maps using custom modifications of functions for visualising EEG brain network provided via Matlab^®^ Central File Exchange (Johann, n.d.).
(6) We also estimated GC between all 6 EEG channels and ECG at wakefulness and each sleep stage, to characterise how brain-cardiovascular relationships differ during wakefulness and sleep.

## Results

The GC of the EEG and ECG time series was estimated for 50 randomly chosen participants. Our random sample comprised 33 male and 17 female subjects (maintaining the 2/3 male, 1/3 female ratio of the initial training dataset), with mean age 53.5 years of age (standard deviation 12.3). Across all 50 subjects, 4.3% of the data were excluded from the analysis due to non-stationarity. The remaining 95.7% of data that were available for the analysis comprised a total of 363.8 h.

**FIGURE 1:**
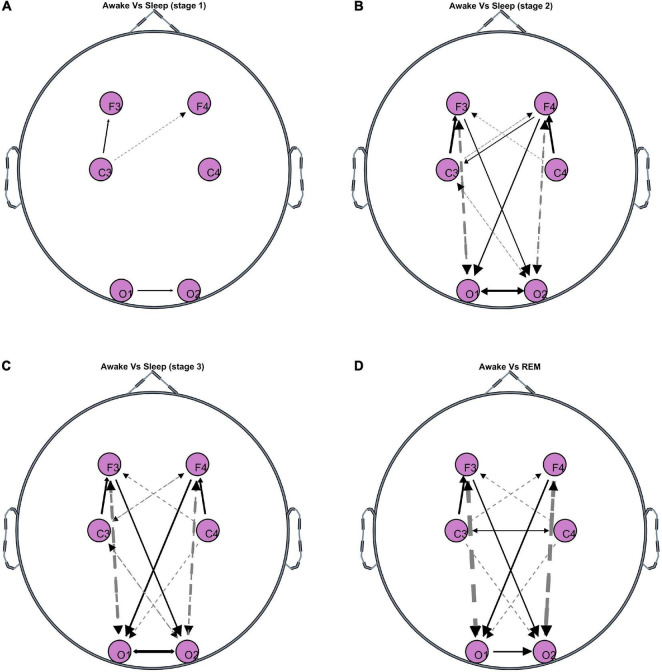
Topographies of significant GC differences between wakefulness and sleep [**(A)** Stage 1, **(B)** stage 2, **(C)** stage 3, **(D)** REM], at F3, F4, C3, C4, O1, and O2 electrode locations. Results are averaged over all 50 participants. Dashed lines indicate negative differences, i.e., GC during the specific sleep stage is significantly greater than GC during wakefulness. Solid lines indicate positive differences, i.e., GC during the specific sleep stage is significantly lower than GC during wakefulness. The line thickness corresponds to the strength of the difference, i.e., thicker lines represent larger differences compared to thinner lines.

Figure 1 shows the significant differences in GC between wakefulness and all sleep stages. The line thickness is proportional to the value of the GC difference (thicker lines represent larger differences). Dashed (solid) lines represent negative (positive) differences, i.e., GC during wakefulness is lower (higher) than GC during the specific sleep stage. As expected, wakefulness and light sleep have very few significant differences in the GC patterns. However, as the state moves progressively from wakefulness to deeper sleep and REM sleep, there are significant differences in bidirectional fronto-posterior GC. There appears to be a sustained positive GC difference (i.e., decrease during sleep compared to wakefulness) from frontal to posterior areas contralaterally, whose strength remains approximately constant in both NREM and REM sleep compared to wakefulness. In contrast, a negative (i.e., increase during sleep compared to wakefulness) bidirectional fronto-posterior GC difference is observed ipsilaterally, with the strength of this difference increasing with deepening NREM stage, and reaching a maximum during REM sleep. Smaller, but still significant, contralateral increases are also observed between centro-posterior connections, mostly in the direction central→posterior. An interesting observation is the significant decrease in motor connectivity (C3-C4), which is distinct for REM sleep stage compared to wakefulness.

**FIGURE 2:**
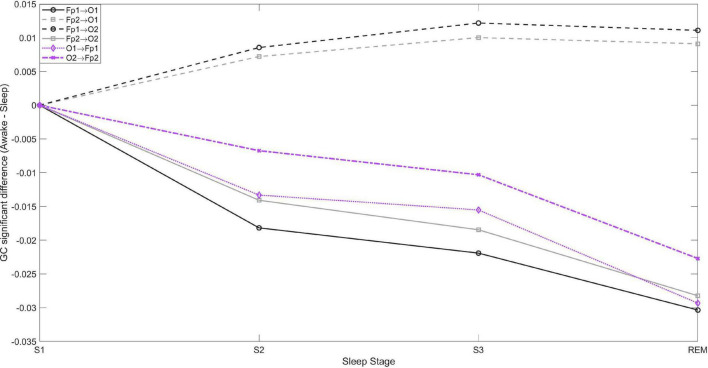
Fronto-posterior significant average GC differences between wakefulness and sleep stages S1, S2, S3, and REM. Negative values indicate GC is greater during sleep compared to wakefulness, and *vice versa*. In the posterior→frontal direction only the contralateral GC was significant, while in the fronto→posterior direction both contralateral and ipsilateral connectivity were significant.

Even though the increase in brain GC during sleep is bidirectional, the increase is larger in the fronto→posterior direction. This is shown in Figure 2, which depicts the differences in fronto-posterior interactions during each sleep stage compared to wakefulness. Negative (positive) values indicate increase (decrease) of GC during the specific sleep stage compared to wakefulness. Thus, bidirectional ipsilateral fronto-posterior GC increases progressively during sleep, but increase is larger in the fronto→posterior direction. In contrast, a progressive decrease is observed contralaterally in fronto→posterior GC, with corresponding connectivity in the opposite direction not being significant.

**FIGURE 3:**
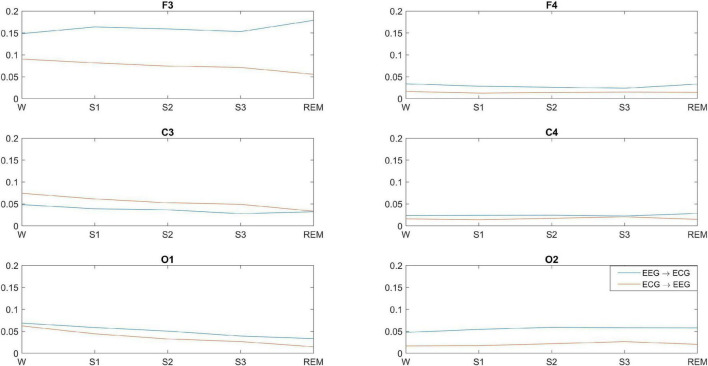
Significant average GC between brain-heart at different EEG locations and across wakefulness and sleep. GC is significant in both directions, but the dominant direction of interaction is brain→heart, with the exception of C3.

Figure 3 shows the significant GC between each EEG channel and ECG for wakefulness and sleep. There is significant bidirectional EEG-ECG causality for all sleep stages and wakefulness, with GC(F3-ECG) having a much larger value compared to all other GC values. With the exception of C3-ECG, GC is higher in the direction EEG→ECG for wakefulness, NREM and REM sleep. There is a clear pattern of GC over the left hemisphere and ECG, compared to the right hemisphere. The GC shows a progressive decrease from wakefulness through to lighter and then deeper NREM stages, and is lowest for REM sleep. This pattern is more prominent in the direction ECG→EEG. No clear pattern can be discerned for EEG-ECG connectivity over the right hemisphere.

## Discussion

*Fronto-posterior EEG connectivity decreases contralaterally, but not ipsilaterally, during sleep.* Wakefulness and light sleep have very few significant differences in GC patterns. This is expected as NREM stage 1 sleep is a transition period from wakefulness. Even though the brain and heart activity begin to slow down during stage 1 sleep, the brain activity still resembles that of a relaxed but awake state, and people who are woken up during stage 1 sleep will often report that they have not been asleep. The increasing negative difference in ipsilateral fronto-posterior GC indicates that GC increases progressively as we move into deeper NREM stages and subsequently into REM sleep. This implies that, ipsilaterally, not only there is no disconnection between frontal and posterior regions during sleep, but there is an increase in their bidirectional connectivity progressively from stages 1–2 to deep sleep and to REM sleep. However, the increase is larger in the fronto-posterior direction compared to the posterior-frontal direction. In contrast, the positive sustained difference contralaterally from frontal to posterior regions, as well as over the occipital cortices, suggests some form of disconnection (decreased connectivity) between the two hemispheres during sleep compared to wakefulness. The strength of this disconnection appears to be independent of the sleep stage. These selective increases and decreases in synchronized activity of specific brain structures are thought to be driving the EEG activity patterns observed during REM sleep (Reinoso-Suárez et al., 2001). Despite the work of Faes et al. also applying GC to brain and heart time series obtained during sleep from healthy subjects, direct comparison with our findings is non-trivial: Faes et al. present GC strength between different frequency ranges across all EEG locations (Faes et al., 2015, 2014). However, they too report significant information transfer during whole-night polysomnographic recordings, both within the brain network and the brain-heart system.

Different methods of connectivity can contribute complementary information to the field. For example, similar ipsilateral increase in fronto-posterior connectivity is reported by Salih et al., who studied EEG connectivity (coherence and Directed Transfer Function) during awake and non-REM sleep (Salih et al., 2009). These findings are also in line with those reported by Massimini et al., and de Gennaro et al., describing synchronizing oscillations that, during sleep, sweep the cortex in the anterior to posterior direction, and which have an important role in wakefulness-sleep transition (De Gennaro et al., 2004; Massimini et al., 2004). Specifically, in de Gennaro et al., changes in bidirectional fronto-posterior Directed Transfer Function (DTF) during transition to sleep (De Gennaro et al., 2004). Namely, an increase in frontal to parieto-occipital DTF is observed at sleep onset, compared to the pre-sleep state. The authors also report a decrease in corresponding parieto-occipital to frontal DTF. In both cases the changes are observed across all frequency ranges studied (δ/θ, α, σ, β). The EEG channels used by de Gennaro et al. are along the midline (Fz, Pz, and Oz), hence we cannot directly compare with our own work that captures changes across the left and right hemispheres. We also report changes across all sleep stages, as opposed to the wake-sleep transition. Related work by Salih et al. shows bidirectional fronto-posterior connectivity that is sustained during sleep, with connectivity in posterior→frontal direction increasing from light to deep sleep, and decreasing in the opposite direction (Salih et al., 2009). Despite the above, frontal→posterior connectivity remains higher compared to the opposite direction.

Another complementary work is by Bartsch et al., who report Time Delay Stability (non-directional connectivity measure based on time-delayed correlation) in brain-brain, as well as brain-heart networks during sleep (Bartsch et al., 2015). The authors report brain-brain interactions of similar topology during wakefulness and light sleep, cross-hemisphere interactions (Fp1-O2, Fp2-O1) that are not as pronounced during deep sleep and REM sleep, decrease in O1-O2 connectivity during sleep compared to wakefulness, and lower C3-C4 connectivity during REM compared to wakefulness. However, there are also some important differences, such as the ipsilateral fronto-posterior connectivity, which Bartsch et al., report as decreasing during light and deep sleep, and increasing during REM sleep but still remaining at a lower level compared to wakefulness. This is in contrast to our findings, which show a sustained increase in ipsilateral bidirectional fronto-posterior connectivity (Figure 3). This can be interpreted in a complementary manner when considering that the TDS measure is based on nondirectional correlation, while the GC measure we use is causal and bidirectional and, thus, more specific (correlation is not causation). Another difference is that TDS captures time delays at which correlation remains at its maximum for a number of consecutive time windows, while GC captures causal connections at zero time lag only.

The differences in ipsilateral and contralateral connectivity observed during sleep could be interpreted in terms of the Default Mode Network (DMN), a circuit of brain regions that is highly active in the absence of overt behavior or in the absence of cognitively demanding tasks. During light sleep, DMN connectivity persists in the form of frontal-posterior coherence. As we progress into sleep, and particularly at deep sleep stages, there is decoupling of frontal areas and a reduction in frontoparietal correlation, suggesting that coherent activation of all parts within the network leads to a conscious experience (Horovitz et al., 2009). We have also observed such fronto-posterior decoupling, contralaterally but not ipsilaterally. Despite the lowest level of logical thinking in REM sleep, connectivity between different parts of the brain was found to be stronger than in NREM sleep; again, this is something that we have also observed ipsilaterally. This supports the hypothesis that connectivity patterns involving DMN subsystems may reflect the underlying brain function in REM sleep (Koike et al., 2011).

In addition, it has long been postulated that wakefulness and REM sleep are fundamentally equivalent functional states, but differ in the way that sensory information and cortical inhibition are handled (Llinás and Paré, 1991). The differences identified in the connectivity between wakefulness and REM sleep in this study may provide some insight to the underlying connectivity mechanisms that set these two functionally equivalent states of wakefulness and REM sleep apart. Another interesting observation is the significant decrease in motor (C3-C4) connectivity observed during REM, in contrast to wakefulness and non-REM sleep stages, during which no significant change in connectivity over the motor cortex is observed. This could be related to the muscle atonia that is specific to REM sleep stage.

*EEG-ECG connectivity decreases as sleep deepens, but increases during REM, with more pronounced changes over the left hemisphere.* Our findings indicate that bidirectional brain-heart GC decreases progressively from wakefulness to sleep, while in some cases GC during REM is stronger than other sleep stages, but still lower than wakefulness. However, there are two interesting observations from our findings. The first one is that, while the dominant direction of connectivity we observe is from brain→heart at all electrode pairs, this does not hold for the C3-ECG pair, where the connectivity is strongest in the ECG→C3 direction. The second interesting observation is the strength of GC for the F3-ECG pair, which is strongest than all other brain-heart connections, and also displays a distinct (and reversed) pattern of F3→ECG connectivity compared to all other brain locations. GC(F3ECG) increases from wakefulness to light sleep (S1), decreases progressively in stages S2 and S3, and then increases again during REM at a level that is even higher than wakefulness. The GC measure we used captures GC patterns across the entire frequency content of the time series analysed, but it is likely that the observed patterns could be related to specific frequency rhythms of the EEG and ECG signals during the different sleep stages. For example, in the study by Faes et al., a strong bidirectional interaction is identified between the high frequency component of heart rate variability and EEG β power, and a weaker unidirectional connection is identified from heart to brain at slower EEG rhythms (Faes et al., 2014).

It has always been the conventional belief that brain commands the heart, but in recent years this has been reconsidered, and it has also been shown that the cardiac system communicates with the brain not only through one but rather through multiple brain rhythms simultaneously. Studies report that, synchronized to the activity of the heart, there is a significant amount of the alpha brain rhythm (Wölk and Velden, 1989), as well as the beta brain rhythm (McCraty et al., 2009). In addition, different branches of the autonomic nervous system are more active during sleep: the sympathetic branch of the autonomic system is more active during wakefulness and REM, while parasympathetic control is more dominant during non-REM sleep (Baharav et al., 1995). Changes in the neural regulation of cardiac dynamics during different sleep stages (in healthy subjects) have also been reported by Lin et al. (2016). Bidirectional interactions have been identified with positive correlation in the brain-heart direction being higher than the negative (heart-brain) direction. The authors also identify that heart→brain interaction is maximum at a time delay of approximately 6 s in light and deep sleep, which decreases in REM and vanishes in wakefulness.

Bartsch et al. have shown that different brain rhythms mediate brain-heart communication during sleep (Bartsch et al., 2015). They also show that the brain-heart network is characterized by relatively symmetric links strength to all six brain areas during wakefulness and sleep, but with stronger links during wakefulness and light sleep, and weaker links during deep sleep and REM [this is also shown in Schmitt et al. (2009)]. Specifically, the authors report that ‘the average link strength for the entire network of brain-heart interactions is highest during W [wakefulness] and LS [light sleep], lower during REM and lowest during DS [deep sleep]’, as obtained via the non-directional correlation-based measure of TDS (Bartsch et al., 2015). This is mostly in agreement with our findings, despite the difference in connectivity measures used in the two studies.

*Limitations.* Despite the popularity of GC use in neuroscience applications, there are a number of known limitations specific to the method (Stokes and Purdon, 2017). GC is, by definition, a linear measure of causality, therefore the connectivity patterns discussed in this work reflect linear interrelationships between brain-brain and brain-heart during wakefulness and sleep. It is possible that a non-linear measure of GC (e.g., Nicolaou and Constandinou, 2016) may capture additional connections that have not been identified in this study or related sleep studies that use other linear measures of connectivity (e.g., correlation, coherence, DTF). This is not unlikely as some studies report that the type (i.e., linear or non-linear) of brain-heart dynamics may show dependence on the frequency range studied (Dumont et al., 2004). Another consideration with the use of connectivity methods in EEG analysis is the potential spurious causality / connectivity that can arise if not all the variables are included in the model, or if there are some latent variables. To eliminate this problem, in theory one must include all sources of influence into the estimation, which is practically unfeasible. As a result, connectivity methods will always be provisional, but the extension to multivariate models, such as the one used in this study, provides an intermediate solution as at least all information that is available is utilized in the GC estimation. Lastly, a general limitation of EEG connectivity analyses is the nature of the scalp-recorded EEG, which are a mixture of attenuated activity from various brain sources as well as potential volume conduction artifacts. Hence, when conducting such analyses, the observed connectivity patterns should be interpreted in reference to the EEG electrode locations, which should not be used as a proxy to underlying brain areas (though one can hypothesize based on the physiological mechanisms that characterize the specific states being studied). One way of minimizing volume conduction or extracting some source information from surface EEG is through source decomposition, e.g., using a state-space (Manomaisaowapak et al., 2022) or Independent Component Analysis (Cohen and Mohammad-Rezazadeh, 2015) approach, and subsequent estimation of connectivity from the decomposed signals. However, even such decomposition does not pinpoint the location of the source, thus, the estimated directed connectivity between the reconstructed sources is not always a reflection of the ground truth (Anzolin et al., 2019). Hence, the interpretation of GC of such sources as functional connectivity between brain regions is as problematic as for sensor level GC. In addition, such methods rely on having an accurate head model and sufficient number of EEG signals (in this study there are only 6 EEG channels available) to be able to capture the underlying brain sources with higher accuracy. An indication that findings are due to spurious connectivity from volume conduction is very strong connectivity at neighboring electrodes, whose strength decreases with increasing inter-electrode distance. Looking at our findings (Figure 1), we detect the presence of connectivity between fronto-posterior electrodes (long distance apart), which is stronger than the connectivity observed at neighboring electrodes.

## Conclusions

The application of GC to study brain-brain and brain-heart bidirectional connections during wakefulness and sleep confirmed the importance of fronto-posterior connectivity during these two states, but has revealed differences in ipsilateral and contralateral mechanisms of these connections. It has also confirmed the existence of bidirectional brain-heart connections that are stronger from brain to heart, with the exception of the left central brain C3-heart connection, which is stronger in the heart-to-brain direction. Given the relationship between the variation in autonomic nervous system (ANS) activation during different sleep stages, future work could include complementary characterization of the two ANS branches (sympathetic and parasympathetic) during wakefulness and sleep with, for example, Poincaré plots (Ardissino et al., 2019).

## Data availability statement

Publicly available datasets were analyzed in this study. This data can be found here: https://physionet.org/content/challenge-2018/1.0.0/.

## Author contributions

HA, MD, SP, and NP: writing and revising of manuscript. SN: reviewing of results and reviewing of manuscript. NN: conception of the work, data analysis, preparation and reviewing of results and writing and revising of manuscript. All authors contributed to the article and approved the submitted version.
